# Efficacy and safety of successful mechanical thrombectomy in elderly patients with anterior circulation large vessel occlusion acute ischemic stroke

**DOI:** 10.3389/fneur.2025.1646081

**Published:** 2025-09-10

**Authors:** Wensheng Zhang, Wen Lin, Yangchun Wen, Haiping Lan, Minzhen Zhu, Xiaojing Zhong, Weifang Xing, Zhenqin Jiang

**Affiliations:** ^1^Department of Neurology, Heyuan People’s Hospital, Guangdong Provincial People's Hospital Heyuan Hospital, Heyuan, China; ^2^Heyuan Key Laboratory of Molecular Diagnosis & Disease Prevention and Treatment, Doctors Station of Guangdong Province, Heyuan People's Hospital, Heyuan, China; ^3^Department of Brain Disease, Maoming Traditional Chinese Medicine Hospital, Maoming, China; ^4^Department of Neurology, Heyuan Hospital of Traditional Chinese Medicine, Heyuan, China

**Keywords:** anterior circulation, large vessel occlusion, acute ischemic stroke, advanced age, mechanical thrombectomy

## Abstract

**Objective:**

To explore the efficacy and safety of successful mechanical thrombectomy (MT) in elderly patients with anterior circulation large vessel occlusion acute ischemic stroke (AIS).

**Methods:**

A retrospective analysis was conducted on 437 patients admitted to our hospital from January 2019 to November 2023 who received successful MT because of anterior circulation large vessel occlusion AIS. They were divided into an elderly group (age ≥ 80 years old) and a young group (age<80 years old). Their clinical data were analyzed.

**Results:**

There were 61 cases in the elderly group and 376 cases in the young group. Before and after propensity score matching analysis, there was no significant difference in any type of cerebral hemorrhage, cerebral hernia, futile recanalization and all-cause mortality between two groups of patients with a *p* value greater than 0.05.

**Conclusion:**

The incidence of any type of cerebral hemorrhage, cerebral hernia, futile recanalization and all-cause mortality in elderly patients with anterior circulation large vessel occlusion AIS was similar to that in young patients. Compared to young patients, MT is also effective and safe in elderly patients.

## 1 Introduction

AIS with large vessel occlusion has a high rate of mortality and disability (1). MT had been proved to be one of the most effective methods for treating large vessel occlusion AIS (2). With the increasing aging of the population, about 30% of patients with AIS with anterior circulation large vessel occlusion were older than or equal to 80 years old (3). As the time window for MT treatment was further extended (4, 5), more elderly patients with AIS with large vessel occlusion will receive MT treatment (6). Previous studies had shown that compared to young patients, elderly patients had higher mortality rates and more severe disability situations (7, 8). However, it had been proved that advanced age was not an independent predictor of adverse outcomes (9). In different studies, there are differences in the efficacy of MT in elderly patients with acute cerebral infarction caused by occlusion of large blood vessels of anterior circulation, which may be related to factors such as different underlying diseases and physiological conditions of the included patients. It can be said that there is still controversy over the efficacy and safety of MT in elderly patients with acute cerebral infarction caused by large vessel occlusion. In this study, we aimed to explore the difference in efficacy and safety between elderly and young patients with AIS caused by anterior circulation large vessel occlusion and received successful MT.

## 2 Materials and methods

### 2.1 Research object

**Figure 1:**
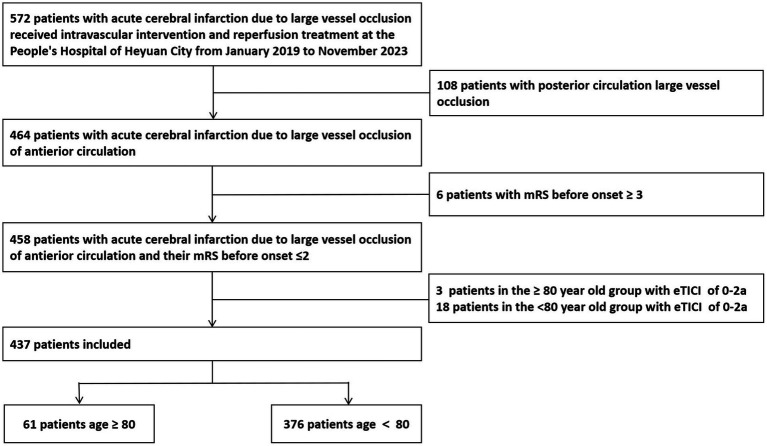
Study flowchart. mRS, modified Rankin scale.

We retrospectively analyzed the clinical data of 437 patients with AIS with anterior circulation occlusion and received successful MT in our hospital from January 2019 to November 2023. Inclusion criteria: (1) Baseline National Institutes of Health Stroke Scale (NIHSS) score at admission ≥ 6 points; (2) At admission, the Alberta Stroke Program Early CT (ASPECT) score ≥ 6 points; (3) After evaluation by cerebral CT, head and neck CTA, or cerebral magnetic resonance imaging, the time from onset to femoral artery puncture ≤ 24 h; (4) The oclusion of large blood vessels was confirmed in the anterior circulation by computed tomography angiography (CTA); (5) The patient or family member signed and agreed to receive MT treatment and did not know if the patient will be included in this study; and (6) MT was successful, with the expanded Thrombolysis in Cerebral Infarction (eTICI) grading of forward blood flow after intervention recanalization 2b-3. Exclusion criteria: (1) Previous modified Rankin scale (mRS) score ≥3 point; (2) MT failure, eTICI grading of forward blood flow was 0–2a; and (3) The patient or family member refused to receive MT treatment. The Medical Ethics Committee of Heyuan People’s Hospital approved our study and all methods were performed in accordance with the relevant guidelines and regulations. The research flowchart is shown in Figure 1.

### 2.2 Patient data collection

We collected basic information about patients, including age, gender, medical history, NIHSS score at onset, mRS score before onset, ASPECT score at admission, whether intravenous thrombolysis was performed, occluded site, American society of intervention and therapeutic neuroradiology/Society of interventional radiology (ASITN/SIR) collateral circulation score (10), eTICI grading, onset to recanalization time, puncture to recanalization time, Trial of Org10172 in Acute Stroke Treatment (TOAST) classification. The site of vessel occlusion can be divided into 5 categories: M1 segment of the middle cerebral artery, M2 segment of the middle cerebral artery, internal carotid artery, anterior cerebral artery, and anterior circulation tandem occlusion. We evaluated collateral circulation using the ASITN/SIR scoring system using DSA imaging (10). The degree of vessel recanalization was evaluated using the eTICI vascular recanalization grade, with eTICI grade ranging from 2b to 3 defined as successful recanalization. The TOAST classification can be divided into 3 categories: atherosclerosis, cardiogenic and other causes. The definition of futile recanalization was successful recanalization of occluded vessel, but the patient still could not achieve independent neurological function at 3 months after MT, and the mRS score > 2 points (11). And we collected information on whether patients suffered from complications such as symptomatic cerebral hemorrhage, asymptomatic cerebral hemorrhage, subarachnoid hemorrhage, cerebral hernia, stroke associated pneumonia, deep vein thrombosis, gastrointestinal bleeding, and other complications. Among them, symptomatic cerebral hemorrhage refers to the occurrence of hemorrhage after MT in patients and an increase in NIHSS score ≥ 4 points (12). Cerebral hernia refers to the displacement of the midline of the patient’s cerebral tissue and the unequal diameter of the bilateral pupils after MT (13).

### 2.3 Follow up and prognostic assessment of patients

At 3 months after MT, we followed up the patients by telephone and the neurological function of patients was evaluated using mRS score, with a score of 0–2 indicating effective recanalization, 3–6 indicating futile recanalization, 0–1 indicating excellent prognosis and 6 indicating death (14, 15). The primary endpoint of this study was the prognosis at 3 months after MT.

### 2.4 Statistical processing

Our study used SPSS 26.0 software to perform statistical analysis on the data. If the quantitative data conformed to a normal distribution, data was expressed as mean ± standard deviation (x ± s), and independent sample *t*-test was used for inter group comparison. Non normal distribution was described with median and interquartile intervals, and rank sum test was used for inter group comparison. Count data was expressed in percentage, and intergroup comparisons were conducted using chi square or Fisher’s exact test. A *p*-value <0.05 was considered statistically significant.

## 3 Results

### 3.1 Comparison of baseline data between elderly and young group of patients

Before propensity matching, the gender, history of atrial fibrillation, smoking history, NIHSS score at onset, time from onset to recanalization of occluded vessel, and TOAST classification of two groups of patients were statistically significant (*p* < 0.05). The baseline data of two groups of patients are shown in Table 1.

**Table 1:** Clinical baseline data of elderly and young patients who received successful MT treatment.

Variables	Elderly group (*n* = 61)	Young group (*n* = 376)	*p* value
Gender, male, *n* (%)	26 (42.62)	273 (72.61)	**<0.001**
Medical history			
Hypertension, *n* (%)	37 (60.66)	200 (53.19)	0.278
Diabetes, *n* (%)	10 (16.39)	68 (18.09)	0.749
Cerebral infarction, *n* (%)	9 (14.75)	60 (15.96)	0.811
Coronary heart disease, *n* (%)	10 (16.39)	42 (11.17)	0.243
Atrial fibrillation, *n* (%)	18 (29.51)	61 (16.22)	**0.012**
Smoking, *n* (%)	13 (21.31)	164 (43.62)	**0.001**
Drinking, *n* (%)	15 (24.59)	85 (22.61)	0.732
mRS score before onset, (mean ± standard deviation)	0.11 ± 0.37	0.07 ± 0.33	0.147
The situation at the onset of cerebral infarction			
NIHSS score, median (IQR)	14 (11–17)	12 (8–15)	**<0.001**
ASPECT score, median (IQR)	7 (7–8)	8 (7–9)	0.108
Received intravenous thrombolysis treatment, *n* (%)	27 (44.26)	133 (35.37)	0.181
Location of occluded blood vessels, *n* (%)			0.886
M1 segment of middle cerebral artery	30 (49.18)	182 (48.40)	
M2 segment of middle cerebral artery	3 (4.92)	21 (5.59)	
Internal carotid artery	7 (11.48)	55 (14.63)	
Anterior cerebral artery	0 (0)	3 (0.80)	
Anterior circulation tandem lesion	21 (34.43)	115 (30.59)	
Collateral circulation score, median (IQR)	2 (2–3)	3 (2–3)	0.293
eTICI grading of forward blood flow for reperfusion, *n* (%)			0.900
2b	15 (24.59)	85 (22.61)	
2c	3 (4.92)	23 (6.12)	
3	43 (70.49)	268 (71.28)	
Time nodes			
Time from onset to reperfusion, min, median (IQR)	478 (348–595)	557.5 (418.5–778)	**0.007**
Puncture to reperfusion time, min, median (IQR)	92 (73.5–120.5)	96 (71.25–128)	0.651
Pathogenesis TOAST classification			**<0.001**
Atherosclerotic type	19 (31.15)	193 (51.33)	
Cardiogenic	33 (54.10)	85 (22.61)	
Other reasons	9 (14.75)	98 (26.06)	

MT, mechanical thrombectomy; mRS, modified Rankin scale; NIHSS, National Institute of Health stroke scale; ASPECT, Alberta Stroke Program Early CT Score; eTICI, expanded Thrombolysis in Cerebral Infarction; TOAST, trial of Org10172 in acute stroke treatment. When the *p*-value is less than 0.05 and there is a statistical difference, the *p*-value is bolded.

### 3.2 Comparison of complications between elderly and young group of patients

Before propensity matching, the incidence of stroke associated pneumonia and gastrointestinal bleeding in two groups of patients was statistically significant (*p* < 0.05). The ratio of stroke associated pneumonia in elderly patients to young patients is 1.6, and the ratio of gastrointestinal bleeding in elderly patients to young patients is 2.26. The complications of two groups of patients are shown in Table 2.

**Table 2:** Comparison of complications and prognosis between elderly and young patients who received successful MT treatment.

Variables	Elderly group (*n* = 61)	Young group (*n* = 376)	*p* value
Symptomatic cerebral hemorrhage, *n* (%)	7 (11.48)	22 (5.85)	0.102
Asymptomatic cerebral hemorrhage, *n* (%)	5 (8.20)	49 (13.03)	0.287
Subarachnoid hemorrhage, *n* (%)	2 (3.28)	19 (5.05)	0.548
Brain Hernia, *n* (%)	4 (6.56)	23 (6.12)	0.895
Stroke associated pneumonia, *n* (%)	47 (77.05)	181 (48.14)	**<0.001**
Deep vein thrombosis, *n* (%)	1 (1.64)	13 (3.46)	0.454
Gastrointestinal bleeding, *n* (%)	15 (24.59)	41 (10.90)	**0.003**
Futile recanalization (3 months mRS ≤ 2 points), *n* (%)	38 (62.30)	185 (49.20)	0.058
Excellent prognosis (3-month mRS ≤ 1 point), *n* (%)	14 (22.95)	143 (38.03)	**0.023**
3-month mRS score, (mean ± standard deviation)	3.16 ± 1.85	2.57 ± 1.87	**0.014**
Death due to all causes within 3 months, *n* (%)	4 (6.56)	30 (7.98)	0.701

MT, Mechanical thrombectomy; mRS, modified Rankin scale. When the *p*-value is less than 0.05 and there is a statistical difference, the *p*-value is bolded.

### 3.3 Comparison of prognosis between elderly and young group of patients

**Figure 2:**
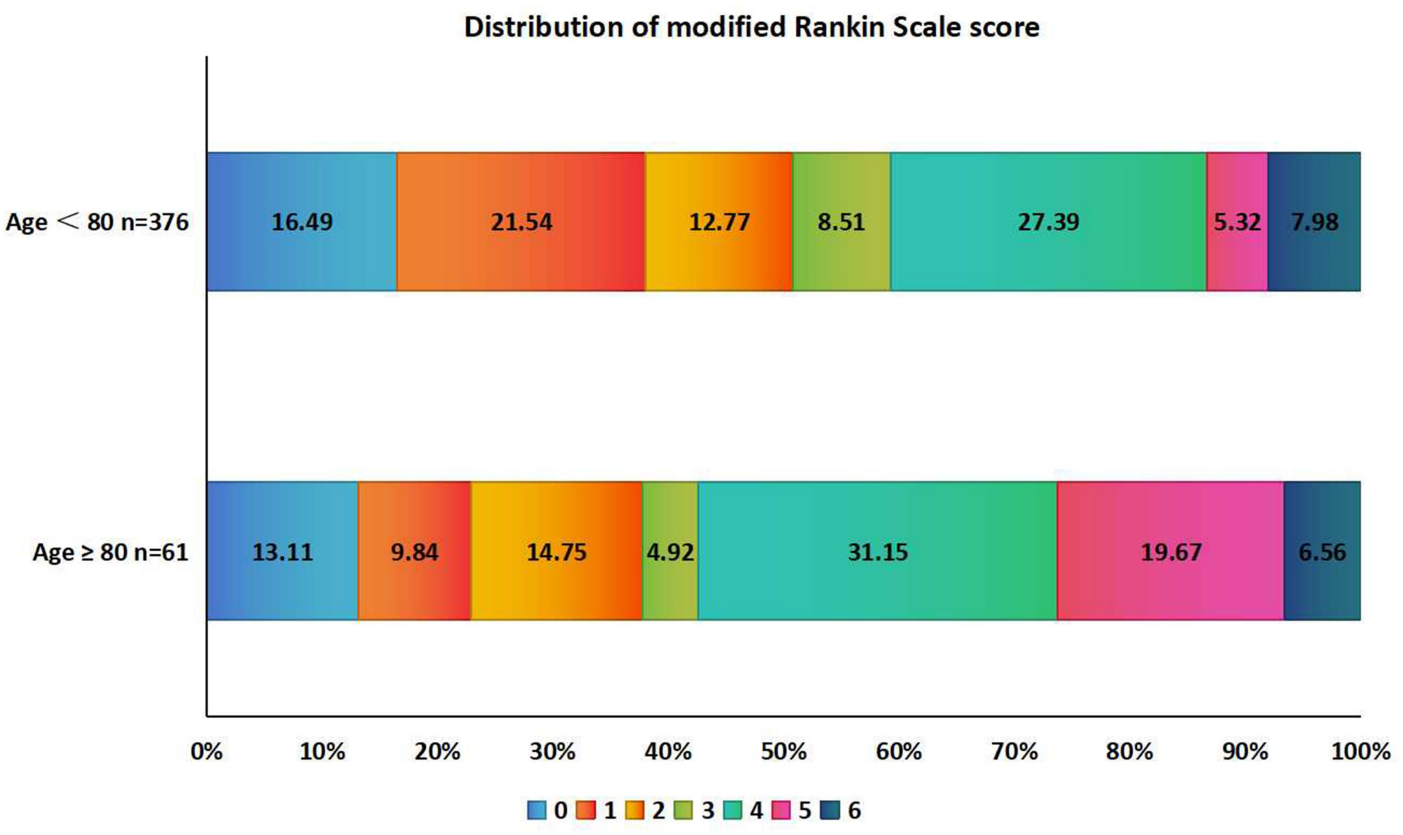
Distribution of mRS score at 3 months after MT in two groups of elderly and young patients. mRS, modified Rankin scale.

The excellent prognosis and mRS score of the two groups of patients at 3 months after onset were statistically significant (*p* < 0.05). The prognosis of two groups of patients was shown in Table 2. The distribution of mRS score 3 months after onset of two groups is shown in Figure 2.

### 3.4 Comparison of complications and prognosis between elderly and young patients who received successful MT treatment after propensity matching

Based on the patients’ gender, atrial fibrillation, smoking status, NIHSS score, and pathogenesis TOAST classification, propensity matching was performed on the elderly and young patient groups, with 34 patients in each group. After propensity matching, there were no statistical differences in complications and prognosis between the elderly and young patients after successful MT. The complications and prognosis of patients after propensity matching are shown in Table 3.

**Table 3:** Comparison of complications and prognosis between elderly and young patients who received successful MT treatment after propensity matching.

Variables	Elderly group (*n* = 34)	Young group (*n* = 34)	*p* value
Symptomatic cerebral hemorrhage, *n* (%)	4 (11.76)	2 (5.88)	0.393
Asymptomatic cerebral hemorrhage, *n* (%)	3 (8.82)	8 (23.53)	0.100
Subarachnoid hemorrhage, *n* (%)	2 (5.88)	1 (2.94)	0.555
Brain Hernia, *n* (%)	1 (2.94)	3 (8.82)	0.300
Stroke associated pneumonia, *n* (%)	25 (73.53)	19 (55.88)	0.128
Deep vein thrombosis, *n* (%)	0 (0)	2 (5.88)	0.151
Gastrointestinal bleeding, *n* (%)	9 (26.47)	7 (20.59)	0.567
Futile recanalization (3 months mRS ≤ 2 points), *n* (%)	12 (35.29)	16 (47.06)	0.324
Excellent prognosis (3-month mRS ≤ 1 point), *n* (%)	8 (23.53)	8 (23.53)	1.000
3-month mRS score, (mean ± standard deviation)	2 (5.88)	3 (8.82)	0.642
Death due to all causes within 3 months, *n* (%)	3.15 ± 1.88	2.79 ± 1.75	0.328

MT, mechanical thrombectomy; mRS, modified Rankin scale.

### 3.5 Comparison of clinical baseline data among elderly patients with effective and futile recanalization

Statistical analysis was conducted on the baseline data of elderly patients using whether the mRS score was ≤ 2 at 3 months after onset as the dependent variable. The ASPECT score, collateral circulation score, eTICI grade, and time from onset to recanalization of occluded vessel were statistically significant (*p* < 0.05) in two groups of patients. The clinical baseline data comparison among elderly patients with effective and futile recanalization is shown in Table 4.

**Table 4:** Clinical baseline data of effective recanalization and futile recanalization in elderly patients.

Variables	Effective recanalization group (*n* = 23)	Futile recanalization group (*n* = 38)	*p* value
Age, years, (mean ± standard deviation)	83.96 ± 3.56	84.08 ± 2.89	0.648
Gender, Male, *n* (%)	7 (30.43)	19 (50)	0.134
Medical History			
Hypertension, *n* (%)	14 (60.87)	23 (60.53)	0.979
Diabetes, *n* (%)	3 (13.04)	7 (18.42)	0.582
Cerebral infarction, *n* (%)	4 (17.39)	5 (13.16)	0.651
Coronary heart disease, *n* (%)	4 (17.39)	6 (15.79)	0.870
Atrial fibrillation, *n* (%)	4 (17.39)	14 (36.84)	0.106
Smoking, *n* (%)	2 (8.70)	11 (28.95)	0.061
Drinking, *n* (%)	4 (17.39)	11 (28.95)	0.310
mRS score before onset, (mean ± standard deviation)	0.17 ± 0.39	0.08 ± 0.36	0.142
The situation at the onset of cerebral infarction			
NIHSS score, median (IQR)	13 (12–19)	14.5 (10.75–16.25)	0.940
ASPECT score, median (IQR)	9 (8–9)	7 (6–7)	**<0.001**
Received intravenous thrombolysis treatment, *n* (%)	11 (47.83)	16 (42.11)	0.663
Location of occluded blood vessels, *n* (%)			0.740
M1 segment of middle cerebral artery	13 (56.52)	17 (44.74)	
M2 segment of middle cerebral artery	1 (4.35)	2 (5.26)	
Internal carotid artery	3 (13.04)	4 (10.53)	
Anterior cerebral artery	0 (0)	0 (0)	
Anterior circulation tandem lesion	6 (26.09)	15 (39.47)	
Collateral circulation score, median (IQR)	3 (3–3)	2 (2–2)	**<0.001**
eTICI grading of forward blood flow for reperfusion, *n* (%)			**0.015**
2b	1 (4.35)	14 (36.84)	
2c	1 (4.35)	2 (5.26)	
3	21 (91.30)	22 (57.89)	
Time nodes			
Time from onset to reperfusion, min, median (IQR)	380 (293–516)	496 (410–675.75)	**0.030**
Puncture to reperfusion time, min, median (IQR)	75 (57–100)	101 (76.5–160.5)	0.080
Pathogenesis TOAST classification			0.880
Atherosclerotic type	8 (34.78)	11 (28.95)	
Cardiogenic	12 (52.17)	21 (55.26)	
Other reasons	3 (13.04)	6 (15.79)	

mRS, modified Rankin scale; NIHSS, National Institute of Health stroke scale; ASPECT, Alberta Stroke Program Early CT Score; eTICI, expanded Thrombolysis in Cerebral Infarction; TOAST, Trial of Org10172 in Acute Stroke Treatment. When the *p*-value is less than 0.05 and there is a statistical difference, the *p*-value is bolded.

### 3.6 Comparison of complications among elderly patients with effective or futile recanalization

The incidence of symptomatic cerebral hemorrhage and stroke associated pneumonia in effective and futile recanalization two groups among elderly patients was statistically significant (*p* < 0.05). The comparison of complications between effective and futile recanalization two groups among elderly patients is shown in Table 5.

**Table 5:** Comparison of complications in elderly patients with effective and futile recanalization after successful MT treatment.

Variables	Effective recanalization group (*n* = 23)	Futile recanalization group (*n* = 38)	*p* value
Symptomatic cerebral hemorrhage, *n* (%)	0 (0)	7 (0)	**0.029**
Asymptomatic cerebral hemorrhage, *n* (%)	1 (4.35)	4 (10.53)	0.394
Subarachnoid hemorrhage, *n* (%)	0 (0)	2 (5.26)	0.263
Brain Hernia, *n* (%)	0 (0)	4 (10.53)	0.107
Stroke associated pneumonia, *n* (%)	12 (52.17)	35 (92.11)	**<0.001**
Deep vein thrombosis, *n* (%)	0 (0)	1 (2.63)	0.433
Gastrointestinal bleeding, *n* (%)	4 (17.39)	11 (28.95)	0.31

MT, mechanical thrombectomy. When the *p*-value is less than 0.05 and there is a statistical difference, the *p*-value is bolded.

## 4 Discussion

In our study, we found that before and after propensity score matching analysis, there was no significant difference in any type of cerebral hemorrhage, cerebral hernia, futile recanalization and all-cause mortality between elderly and young two groups of patients.

The situation of aging population was gradually more and more apparent. In patients with AIS caused by large vessel occlusion, the proportion of elderly patients gradually increased (6). Some studies had found that the good prognosis rate of elderly patients with AIS with large vessel occlusion after MT was lower than that of young patients (7, 8), while others had found that the prognosis of elderly patients was not worse than that of young patients (9). Zhou T. et al. (16) found that advanced age was an independent predictor of futile recanalization after endovascular treatment in AIS patients with large vessel occlusion. Given the differences in previous researches findings of MT in elderly patients, we conducted this retrospective study.

The study by Zhao G. et al. (7) found that as age increased, the chances of achieving good prognosis in large vessel occlusion AIS patients who received MT were reduced. Alawieh et al. (8) studied a total of 1,346 patients, including 346 elderly patients and found that the proportion of patients with a good prognosis after 3 months in the ≥ 80 year old group was lower than that in the <80 year old group. The study by Li G. et al. (17) found that age was one of the independent risk factors for predicting poor survival and death within 90 days after mechanical thrombectomy. Our study found that there was no difference in the effective prognosis rate and all-cause mortality rate at 3 months after MT between elderly and young group of patients. This suggested that although elderly patients may have a higher postoperative 3-month mRS score than young patients after successful MT when sufferring from large vessel occlusion AIS, the probability of futile recanalization in elderly patients was not higher than that of young patients. Of course, it required more larger sample size studies to confirm this point.

In terms of complications, some studies suggested that patients aged 80 and above with AIS had poorer physical fitness, recovery ability and neural plasticity compared to young patients (7, 16, 18). In our study, the incidence of stroke associated pneumonia and gastrointestinal bleeding in the elderly group was significantly higher than that of young group before propensity matching. However, after propensity matching, there was no significant difference in any type of cerebral hemorrhage, cerebral hernia, futile recanalization and all-cause mortality between two groups of patients.

In the statistical analysis of elderly patients, it was found that factors affecting the prognosis of patients included ASPECT score at admission, collateral circulation score, eTICI blood flow grading, and the time from onset to recanalization of occluded blood vessel. The lower the ASPECT score at admission, the larger the core infarct volume of the patient, the less salvageable brain tissue, and the greater the possibility of futile recanalization. The higher the collateral circulation score, the larger the ischemic penumbra area that can be salvaged, and the more brain tissue can be salvaged after successful MT. Patients with eTICI blood flow grade 3 have a better prognosis than those with grades 2b-2c. The higher the level of recanalization blood flow, the more sufficient the level of recanalization, which was consistent with previous studies (9). The effective recanalization group had a shorter time from onset to recanalization compared to the futile recanalization group, which suggested that the earlier the occluded cerebral blood vessels were recanalized, the earlier the cerebral ischemia can be relieved, and there would be more surviving brain tissue, resulting in a better prognosis for the patient. Ahn Y et al.’s research confirmed that shortening the time from onset to recanalization of occluded blood vessel can improve the prognosis of elderly patients (19). Our research finding was consistent with those of Ahn Y et al.

Cerebral hemorrhage transformation is one of the serious complications after MT for AIS patients with large vessel occlusion (20). Adcock et al. (6) conducted a cohort study analysis on 42,422 patients who received MT and found that there was no statistically significant difference in the proportion of symptomatic cerebral hemorrhage transformation between the elderly group and the young group of patients. In our study, in elderly patients, the incidence of symptomatic intracerebral hemorrhage was higher in patients with futile recanalization compared to those with effective recanalization, indicating that symptomatic intracerebral hemorrhage was also an important factor affecting the prognosis of elderly patients.

Our previous study showed that failure of early neurological improvement can predict futile recanalization after successful MT in AIS patients with anterior circulation large vessel occlusion tandem lesions (21). This study was different from our previous study in terms of inclusion criteria, important predictors, etc. The novelty of this study was to compare elderly patients with large vessel occlusion who received successful MT treatment with young patients, rather than comparing the efficacy differences between elderly patients who received and did not receive MT treatment. This comparison has not been reported yet. In addition, the purpose and finding of our study are similar to some previous studies, but the difference between our study and previous studies is that we did not include patients who received failed MT. We only compared the efficacy and safety of successful MT between elderly and young patients.

Our study also had some limitations. Firstly, this study was a single center, retrospective study, which was difficult to avoid selection bias and the single-center design may limit the generalizability and widely application of our finding. Secondly, the sample size included in our study was relatively limited. Thirdly, excluding patients with failed MT and baseline mRS ≥ 3 may bias the findings. In future, prospective clinical studies with larger sample sizes are needed to further confirm the efficacy and safety of MT in elderly patients.

## 5 Conclusion

The incidence of any type of cerebral hemorrhage, cerebral hernia, futile recanalization and all-cause mortality in elderly patients with anterior circulation large vessel occlusion AIS was similar to that in young patients. Compared to young patients, MT is also effective and safe in elderly patients.

## Data Availability

The datasets analyzed during this study are not publicly available due to intellectual property rights, but are available from the corresponding author on reasonable request.
